# Rapid Synthesis of Carbon‐Supported Ru‐RuO₂ Heterostructures for Efficient Electrochemical Water Splitting

**DOI:** 10.1002/advs.202414534

**Published:** 2025-01-15

**Authors:** Dingjie Pan, Bingzhe Yu, John Tressel, Sarah Yu, Pranav Saravanan, Naya Sangoram, Andrea Ornelas‐Perez, Frank Bridges, Shaowei Chen

**Affiliations:** ^1^ Department of Chemistry and Biochemistry University of California 1156 High Street Santa Cruz California 95064 USA; ^2^ Department of Physics University of California 1156 High Street Santa Cruz California 95064 USA

**Keywords:** bifunctional, magnetic induction heating, Ru‐RuO_2_ heterostructure, water splitting

## Abstract

Development of high‐performance electrocatalysts for water splitting is crucial for a sustainable hydrogen economy. In this study, rapid heating of ruthenium(III) acetylacetonate by magnetic induction heating (MIH) leads to the one‐step production of Ru‐RuO₂/C nanocomposites composed of closely integrated Ru and RuO₂ nanoparticles. The formation of Mott‐Schottky heterojunctions significantly enhances charge transfer across the Ru‐RuO_2_ interface leading to remarkable electrocatalytic activities toward both hydrogen evolution reaction (HER) and oxygen evolution reaction (OER) in 1 m KOH. Among the series, the sample prepares at 300 A for 10 s exhibits the best performance, with an overpotential of only −31 mV for HER and +240 mV for OER to reach the current density of 10 mA cm⁻^2^. Additionally, the catalyst demonstrates excellent durability, with minimal impacts of electrolyte salinity. With the sample as the bifunctional catalysts for overall water splitting, an ultralow cell voltage of 1.43 V is needed to reach 10 mA cm⁻^2^, 160 mV lower than that with a commercial 20% Pt/C and RuO₂/C mixture. These results highlight the significant potential of MIH in the ultrafast synthesis of high‐performance catalysts for electrochemical water splitting and sustainable hydrogen production from seawater.

## Graphical abstract

Magnetic induction heating of Ru(acac)_3_ is carried out for the ultrafast, one‐step preparation of Ru‐RuO_2_/C heterostructured composites, which exhibit a remarkable electrocatalytic performance toward both hydrogen evolution reaction and oxygen evolution reaction and can be used as bifunctional catalysts for electrochemical water splitting. The performance is markedly better than that with commercial benchmarks.

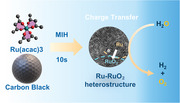

## 1 Introduction

Hydrogen is a unique carrier that can store intermittent solar, wind, and chemical energy through water splitting,^[^ ^1^, ^2^, ^3^ ^]^ which involves hydrogen evolution reaction (HER) at the cathode and oxygen evolution reaction (OER) at the anode. Both require efficient catalysts to achieve sufficiently high current densities for practical applications,^[^ ^4^, ^5^ ^]^ and OER presents significant challenges due to its complex reaction pathways and sluggish electron‐transfer kinetics.^[^ ^6^ ^]^ Prior research has primarily focused on catalysts for the HER or OER half‐reaction, whereas studies remain scarce for bifunctional catalysts.^[^ ^7^, ^8^, ^9^ ^]^ Therefore, developing high‐performance bifunctional catalysts has been recognized as a critical step for the successful implementation and advancement of the technology.

Currently, platinum (Pt) and iridium oxide (IrO_2_) nanoparticles are the benchmark electrocatalysts for HER and OER, respectively.^[^ ^10^, ^11^ ^]^ However, the limited natural abundance and high costs greatly restrict their broad applications. Recently, transition metals, which are far more abundant on the Earth and at much lower costs, have garnered significant interest. Nevertheless, their performance in terms of activity and durability still falls short of commercial standards.^[^ ^12^ ^]^ More affordable noble metals such as ruthenium (Ru) have emerged as promising alternatives. Ru is competitively priced (≈$400 per oz as compared to $987 for Pt and $4700 for Ir) and demonstrates favorable electrocatalytic properties.^[^ ^13^ ^]^ This is mainly due to the similar electronic energy structures of Ru and RuO₂ to those of Pt and IrO₂.^[^ ^14^, ^15^ ^]^

Since metallic Ru demonstrates strong catalytic activity for HER but has limited effectiveness for OER, and RuO₂ excels in OER but is ineffective for HER, the Ru‐RuO₂ combination may exhibit bifunctional activity.^[^ ^16^, ^17^, ^18^, ^19^, ^20^ ^]^ For instance, Wang and coworkers^[^ ^16^ ^]^ prepared graphene composites with Ru‐RuO₂ heterostructures by controlled calcination of RuCl₃, thiourea, and N,P‐codoped reduced graphene oxide nanosheets. The resulting Ru‐RuO₂@NPC nanocomposites demonstrated a remarkable bifunctional activity for both HER and OER across a wide pH range, requiring a low cell voltage (E_10_) of 1.46 V to achieve a current density of 10 mA cm⁻^2^ in electrochemical water splitting. This performance was attributed to charge transfer at the Ru‐RuO₂ Mott‐Schottky (M‐S) junctions, which shifted the d‐band center at the interface to an intermediate position between those of Ru and RuO₂, thus optimizing the adsorption and desorption of key reaction intermediates (e.g., ^*^H, ^*^O, ^*^OH, and ^*^OOH). Ai and coworkers^[^ ^17^ ^]^ developed a robust Ru‐RuO₂ heterostructure by partially oxidizing Ru nanoparticles in amorphous carbon. The strong electronic synergy at the Ru‐RuO₂ interface led to an outstanding acidic OER performance with an ultralow overpotential (η_OER,10_) of +176 mV and excellent stability over 80 h at 10 mA cm⁻^2^. The catalyst was then used as a bifunctional electrocatalyst for overall water splitting, achieving a low E_10_ of 1.55 V with long‐term durability, making it promising for a proton exchange membrane water electrolyzer. Hu and colleagues^[^ ^21^ ^]^ synthesized a porous reticular structure (PRS) Ru/RuO₂ and observed an improved OER performance in both acidic and alkaline media. The Ru/RuO₂‐PRS nanocomposites exhibited a reduced overpotential and enhanced durability by mitigating RuO₂ dissolution at high anodic potentials, effectively addressing major challenges for Ru‐based OER catalysts in acidic environments. In these studies, charge transfer at the Ru‐RuO₂ interface was mostly argued to be responsible for the enhancement of the electrocatalytic performance.

Thus far, such heterojunction samples have been mostly prepared by conventional thermal procedures which were time‐ and energy‐consuming. Recently, magnetic induction heating (MIH) has emerged as an effective procedure for the ultrafast preparation of a range of functional materials.^[^ ^22^ ^]^ MIH takes advantage of the rapid Joule effect to reach temperatures over 1000 °C within seconds at a heating rate up to 200 °C s^−1^, in stark contrast with conventional methods such as tube furnaces and hydrothermal processes, which exhibit a much slower heating rate (<10 °C min^−1^) and typically take hours or even days. Also, the rapid heating and cooling can facilitate the formation of nonequilibrium and metastable structures that are critical for catalysis. For instance, high‐performance OER catalysts have been obtained with FeNi spinel oxides featuring a good mixing of the Fe and Ni phases^[^ ^23^ ^]^ and defective carbon‐encapsulated Co nanoparticle composites,^[^ ^24^ ^]^ while Ru nanoparticles with RuCl*_x_* residues,^[^ ^25^ ^]^ amorphous MoS*_x_* composites,^[^ ^26^ ^]^ and ruthenium nanoparticles/molybdenum oxide/carbon composites^[^ ^27^ ^]^ have been found to possess remarkable electrocatalytic activity toward HER.

In this study, we report the ultrafast preparation of carbon‐supported Ru‐RuO₂ heterostructure catalysts (Ru‐RuO_2_/C) by MIH and observe a high efficiency toward both HER and OER. Such a bifunctional property can then be exploited for electrolysis even in simulated alkaline seawater. Experimentally, ruthenium(III) acetylacetonate (Ru(acac)₃) is used as the sole precursor and undergoes a disproportionation reaction during MIH treatment, forming Ru‐RuO₂ heterostructures due to the oxygen‐rich environment as a result of thermal decomposition of the acac ligands.^[^ ^28^ ^]^ Among the series, the sample prepared at 300 A for 10 s (Ru‐RuO_2_/C‐300A) exhibits the best electrocatalytic activity toward both HER and OER in alkaline media, featuring a low overpotential of −31 and +240 mV to reach the current density of 10 mA cm^−2^, respectively. The sample also possesses excellent durability, with minimal impacts of electrolyte salinity on the electrocatalytic performance. When the sample is used as bifunctional catalysts for full water splitting, an ultralow E_10_ of only 1.44 V is required. The excellent electrocatalytic activity is attributed to the formation of Ru‐RuO_2_ heterostructures that facilitate charge transfer at the M‐S heterojunction and the optimal adsorption of key reaction intermediates.

## 2 Results and Discussion

### 2.1 Sample Preparation and Structural Characterization

The Ru‐RuO_2_/C nanocomposites were prepared by using the MIH apparatus described previously.^[^ ^23^, ^24^, ^25^, ^26^, ^27^ ^]^ Experimentally, Ru(acac)_3_ was used as the sole precursor and loaded onto carbon black, which was then subject to MIH treatment at different induction currents (X = 200–600 A) for 10 s in an argon atmosphere. The obtained samples were referred to as Ru‐RuO_2_/C‐X. The synthetic details are included in the Experimental Section.

**Figure 1:**
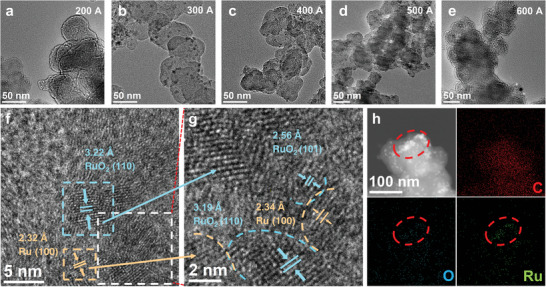
a–e) TEM images of the Ru‐RuO_2_/C‐X samples prepared at different induction currents. f) High‐resolution TEM image of Ru‐RuO_2_/C‐300A and lattice fringe analysis, g) zoom‐in of the white dashed box in panel (f), and h) the corresponding elemental maps.

During MIH heating, Ru(acac)₃ started to decompose at ≈150 °C and underwent a disproportionation reaction,^[^ ^29^ ^]^ where part of Ru^3^⁺ was reduced into metallic Ru, while the other was oxidized by the thermally decomposed acetylacetonate ligands to form RuO₂.^[^ ^28^ ^]^ The structure of the Ru‐RuO_2_/C‐X nanocomposites was first characterized by transmission electron microscopy (TEM) measurements. From **Figures** **1**a and S1 (Supporting Information), the Ru‐RuO_2_/C‐200A sample can be seen to possess only agglomerates of flaky carbon, suggesting that at this low induction current (temperature ≈600 °C), mostly amorphous Ru clusters were produced.^[^ ^25^ ^]^ Yet, with the increase of the induction current (e.g., 300–600 A), dark‐contrast nanoparticles of 5–10 nm in diameter started to emerge, suggesting the formation of Ru‐based nanoparticles (Figure 1b–e). In high‐resolution TEM measurements of the Ru‐RuO_2_/C‐300A sample (Figure 1f and the zoom‐in image in Figure 1g), two sets of well‐defined lattice fringes can be resolved, one with an interplanar spacing of ≈2.34 Å that can be ascribed to the Ru(100) planes, and the other at 2.56 and 3.20 Å in good agreement with those of RuO_2_(101) and (110) planes, respectively.^[^ ^30^, ^31^ ^]^ Furthermore, the intimate contact between the Ru and RuO₂ crystalline domains suggests the formation of Ru‐RuO_2_ M‐S heterojunctions (Figure 1g). Such a structure can also be found with samples prepared at higher induction currents (Figures S2–S4, Supporting Information), which featured an average size of 3–5 nm in diameter (Figure S5, Supporting Information).

Consistent results were obtained in elemental mapping analysis based on energy‐dispersive X‐ray spectroscopy (EDS) (Figure 1h and Figure S6, Supporting Information), where the C, Ru, and O elements can be clearly identified. Notably, Ru and O were distributed discretely on the carbon black support, consistent with the formation of carbon‐supported Ru‐RuO_2_ nanoparticles.

One may notice that in prior studies using RuCl₃ as the precursor,^[^ ^25^, ^32^ ^]^ MIH treatment under comparable conditions yielded mostly metallic Ru nanoparticles and no RuO₂. The fact that RuO_2_ was produced in the present study can be ascribed to the Ru(acac)_3_ precursor, as the thermal decomposition of metal acetylacetonates has been known to produce metal oxides.^[^ ^33^ ^]^ Yet, as MIH treatment lasted only a few seconds, not all metallic ruthenium was oxidized into RuO_2_, leading to the formation of Ru‐RuO_2_ M‐S heterojunctions in the final products.

X‐ray photoelectron spectroscopy (XPS) measurements were then conducted to investigate the surface elemental composition and valence states of the Ru‐RuO_2_/C nanocomposites. From the survey spectra in Figure S7 (Supporting Information), the C 1s/Ru 3d, Ru 3p, and O 1s electrons can be readily identified for all samples at ≈284, 462, and 531 eV, respectively. Based on the integrated peak areas, the samples can be seen to exhibit a rather consistent composition, with ≈80–89 at% of C, 10–13 at% of O, and 0.6–0.9 at% of Ru (Table S1, Supporting Information), in good agreement with results obtained from EDS measurements (Figure S6, Supporting Information).

**Figure 2:**
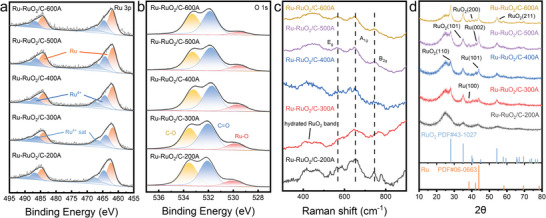
High‐resolution XPS spectra of the a) Ru 3p and b) O 1s electrons, c) Raman, and d) XRD patterns of the Ru‐RuO_2_/C series.

The high‐resolution Ru 3p spectra are depicted in **Figure** **2**a. Ru‐RuO₂/C‐300A can be seen to possess the lowest binding energies among the series, and deconvolution of the data yielded two doublets, the major one at 461.77/483.97 eV due to the 3p₃_/_₂/3p₁_/_₂ electrons of metallic Ru, and the minor one at 463.94/486.14 eV to those of Ru^4+^ (with the associated satellites at 466.75/488.95 eV).^[^ ^34^, ^35^, ^36^ ^]^ In the corresponding O 1s spectra (Figure 2b), three species can be resolved for Ru‐RuO₂/C‐300A at 529.88 eV for lattice oxygen (Ru‐O), 532.03 eV for C═O and 533.51 eV for C─O, consistent with the formation of a Ru and RuO₂ hybrid in the sample.^[^ ^37^ ^]^ The C 1s/Ru 3d spectra are shown in Figure S8 (Supporting Information), where the Ru 3d, C═C, and C─C peaks can be resolved at 280.67/285.16, 284.23, and 284.67 eV, respectively.^[^ ^38^ ^]^ Other samples in the series exhibited similar profiles. Nevertheless, for Ru‐RuO₂/C‐200A, the Ru 3p binding energies were ≈0.5 eV higher than those of Ru‐RuO₂/C‐300A, likely due to the low heating temperature and hence limited decomposition of the Ru(acac)_3_ precursor, leading to the formation of only (amorphous) ruthenium clusters, consistent with results from the TEM measurements (Figure 1a and Figure S1, Supporting Information).^[^ ^25^ ^]^ For samples prepared at higher temperatures (i.e., Ru‐RuO₂/C‐400A, Ru‐RuO₂/C‐500A and Ru‐RuO₂/C‐600A), the binding energies of metallic Ru 3p were only slightly greater than those of Ru‐RuO₂/C‐300A (under 0.2 eV), and an increase up to 0.6 eV was observed with the Ru^4+^ electrons (Table S2, Supporting Information). Such electron depletion was possibly the result of the increasingly oxidizing atmosphere produced during the thermal decomposition of Ru(acac)_3_.^[^ ^33^ ^]^

Table S3 (Supporting Information) lists the metallic Ru and Ru^4+^ contents of the various Ru‐RuO₂/C samples. One can see that among the sample series, Ru‐RuO₂/C‐300A possessed the highest contents of metallic Ru (0.52 at%) and Ru^4+^ (0.41 at%), which are the known active components for HER and OER, respectively (vide infra). The slight decrease observed with Ru‐RuO_2_/C‐400A, Ru‐RuO_2_/C‐500A, and Ru‐RuO_2_/C‐600A likely arose from enhanced thermal vaporization of the precursors.

Raman spectroscopic measurements further confirmed the formation of RuO_2_ in the samples.^[^ ^39^, ^40^ ^]^ From Figure 2c, all samples in the series can be seen to possess a broad peak centered at ≈420 cm^−1^ due to the hydrated RuO_2_ band, which diminished slightly with increasing MIH temperature (from Ru‐RuO₂/C‐200A to Ru‐RuO₂/C‐600A). Three additional bands can be identified. The peak at 561 cm⁻¹ can be assigned to the E_g_ mode of RuO_2_ (out‐of‐plane vibration of Ru‐O), while the bands at 652 and 746 cm⁻¹ can be ascribed to the A_1g_ and B_2g_ modes (in‐plane vibrations of the two O atoms relative to the Ru atom), respectively.^[^ ^41^ ^]^

Further structural insights were obtained from X‐ray diffraction (XRD) measurements (Figure 2d). A broad peak can be observed at 2θ =  25.0° for all samples, characteristic of the (002) planes of graphitic carbon.^[^ ^42^ ^]^ Additional peaks can be found at 2θ =  38.4°, 42.2°, and 44.1° due to the (110), (002), and (101) planes of *hcp* Ru (PDF#06‐0663), respectively, whereas the peaks at 2θ =  27.8°, 35.2°, 40.2°, and 54.3° can be indexed to the (110), (101), (200), and (211) plane of RuO_2_ (PDF#43‐1027).^[^ ^16^ ^]^ Notably, the characteristic peaks for both Ru and RuO₂ were rather broad and ill‐defined for Ru‐RuO_2_/C‐200A but became increasingly sharper with increasing MIH current, indicating enhanced crystallinity of the samples, in good agreement with results from TEM measurements (Figure 1 and Figures S1–S4, Supporting Information).

**Figure 3:**
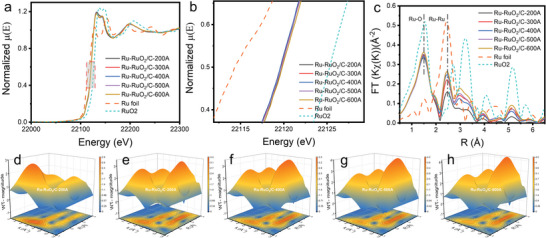
a) Ru K‐edge XANES spectra, b) zoom‐in of the red box in panel (a), and c) Fourier transforms of the Ru K‐edge EXAFS oscillations of the Ru‐RuO₂/C samples and references. The corresponding WT‐EXAFS profiles of d) Ru‐RuO₂/C‐200A, e) Ru‐RuO₂/C‐300A, f) Ru‐RuO₂/C‐400A, g) Ru‐RuO₂/C‐500A, and h) Ru‐RuO₂/C‐600A.

X‐ray absorption spectroscopy (XAS) measurements were then conducted to analyze the Ru coordination environment and electronic structure within the Ru‐RuO₂/C nanocomposites. From the normalized Ru K‐edge X‐ray absorption near‐edge structure (XANES) spectra in **Figure** **3**a,b, the Ru‐RuO₂/C samples can be seen to exhibit a similar absorption edge that lies between those of the Ru foil and RuO₂ references, indicating a rather consistent valence state (between 0 and +4) of the ruthenium centers across the samples. Figure 3c depicts the corresponding R‐space profiles obtained through the Fourier transform of the extended X‐ray absorption fine structure (EXAFS) spectra. All samples can be seen to possess a major peak at 2.47 Å for Ru‐Ru and another one at 1.50 Å for Ru‐O, consistent with the Ru foil and RuO₂ references, respectively, further confirming the formation of a Ru‐RuO₂ M‐S heterojunctions within the samples.

The Ru‐K edge EXAFS data were then fitted using a two‐peak model, and the fitting results are listed in Figure S9 and Table S4 (Supporting Information). The sample series can be seen to display a similar structure, with the Ru─O and metallic Ru─Ru bond lengths at ≈1.96 and 2.68 Å, respectively, in good alignment with those of RuO₂ and Ru foil. Furthermore, the relevant coordination numbers (CN) are all lower than those for the Ru foil (12) and RuO_2_ references (6). In fact, the Ru‐RuO_2_/C‐200A samples exhibited a CN of ≈4.2 for Ru‐C/O and 1.5 for Ru‐Ru, whereas ≈3.8 and 2.6 for others prepared at higher MIH currents. This can be accounted for by the formation of a largely amorphous structure in Ru‐RuO_2_/C‐200A due to insufficient decomposition of Ru(acac)_3_ at the low temperature whereas nanoparticles started to appear at higher temperatures.

The wavelet transform (WT) analysis, shown in Figure 3d–h, yielded consistent results. The analysis was performed using Fortan with the Morlet function.^[^ ^43^, ^44^ ^]^ To ensure comparability of the atomic configurations, all samples were analyzed using the same parameters: κ = 5 and σ = 1. The peaks at 6.4 Å⁻¹ and 10.9 Å⁻¹ correspond to the first and second neighbors of RuO₂, while the peak at 7.3 Å⁻¹ represents the metallic Ru‐Ru bond, again, confirming the formation of a hybrid structure within the composites.

### 2.2 Electrocatalytic Activity

**Figure 4:**
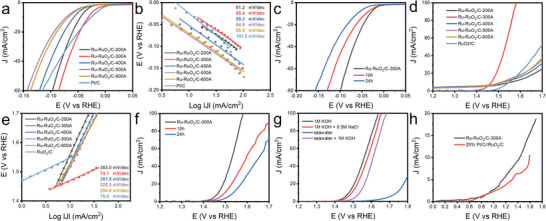
a) HER polarization curves at the rotation rate of 1600 rpm with 100% iR correction and b) the corresponding Tafel plots of the Ru‐RuO₂/C samples in 1 m KOH. c) HER polarization curves of Ru‐RuO₂/C‐300A before and after chronoamperometric (i‐t) tests for 10 and 24 h. d) OER polarization curves at the rotation rate of 1600 rpm and with 100% iR correction and e) the corresponding Tafel plots of the sample series in 1 m KOH. f) Stability tests of Ru‐RuO₂/C‐300A before and after chronoamperometric (i‐t) tests for 10 and 24 h. g) OER polarization curves of Ru‐RuO₂/C‐300A in 1 m KOH, 1 m KON + 0.5 m NaCl, seawater, and seawater + 1 m KOH. h) Current‐potential profiles for full water splitting with Ru‐RuO₂/C‐300A as the bifunctional catalysts or a mixture of Pt/C//RuO_2_/C in 1 m KOH in a two‐electrode system.

The HER and OER electrocatalytic activities of the Ru‐RuO₂/C samples were then evaluated through electrochemical measurements using a standard three‐electrode configuration in 1.0 m KOH (pH = 14). Ru‐RuO₂/C‐300A clearly demonstrated the best HER and OER activity among the series. From the HER polarization curves in **Figure** **4**a, one can see that Ru‐RuO₂/C‐300A required an overpotential (η_HER,10_) only of −31 mV to reach the current density of 10 mA cm⁻^2^, as compared to −46 mV for Ru‐RuO₂/C‐200A, −58 mV for Ru‐RuO₂/C‐400A, −68 mV for Ru‐RuO₂/C‐600A, and −72 mV for Ru‐RuO₂/C‐500A. Such a performance of Ru‐RuO₂/C‐300A is rather comparable to that of commercial Pt/C (−26 mV). Note that at potentials more negative than ≈−50 mV Ru‐RuO₂/C‐300A actually significantly outperformed Pt/C. In fact, the turnover frequency (TOF)^[^ ^45^ ^]^ can be estimated to be 0.67 s⁻¹ at −50 mV for RuO₂/C‐300A, markedly greater than that of Pt/C (0.41 s⁻¹) (Figure S10, Supporting Information).

The corresponding Tafel plots are shown in Figure 4b, where Ru‐RuO₂/C‐300A exhibited a slope of 85.4 mV dec⁻¹, close to other samples in the series but markedly lower than that of Pt/C (101.5 mV dec⁻¹). In fact, from the Nyquist plots in Figure S11 (Supporting Information) acquired at the overpotential of −50 mV, the charge transfer resistance (R_ct_) was indeed relatively close for the series of samples, 24.86 Ω for Ru‐RuO₂/C‐200A, 30.58 Ω for Ru‐RuO₂/C‐300A, 29.98 Ω for Ru‐RuO₂/C‐400A, 32.43 Ω for Ru‐RuO₂/C‐500A, and 28.04 Ω for Ru‐RuO₂/C‐600A.

Moreover, Ru‐RuO₂/C‐300A demonstrated exceptional durability. As shown in Figure 4c and Figure S12 (Supporting Information), the η_HER,10_ shifted negatively by only 11 mV (to −42 mV) after chronoamperometric tests at −31 mV in 1 m KOH for 10 h and by just 29 mV (to −60 mV) after 24 h.

The OER performances of the Ru‐RuO₂/C samples were also tested in 1 m KOH. From the polarization curves in Figure 4d, one can see that Ru‐RuO₂/C‐300A exhibited a low overpotential (η_OER,10_) of +240 mV to achieve 10 mA cm^−2^, as compared to +330 mV for Ru‐RuO₂/C‐200A, +300 mV for Ru‐RuO₂/C‐400A, +280 mV for Ru‐RuO₂/C‐500A, +290 mV for Ru‐RuO₂/C‐600A and +320 mV for commercial RuO_2_/C. In fact, at the overpotential of +300 mV, Ru‐RuO₂/C‐300A possessed a TOF of 1.2 × 10⁻⁴ s⁻¹, substantially higher than that (2.2 × 10⁻⁵ s⁻¹) observed for commercial RuO₂/C (Figure S13, Supporting Information).

The corresponding Tafel plots, shown in Figure 4e, indicate that Ru‐RuO₂/C‐300A possessed the lowest Tafel slope of 74.1 mV dec⁻¹ among the sample series, in comparison to Ru‐RuO₂/C‐200A (363.0 mV dec⁻¹), Ru‐RuO₂/C‐400A (351.5 mV dec⁻¹), Ru‐RuO₂/C‐500A (325.3 mV dec⁻¹), and Ru‐RuO₂/C‐600A (294.6 mV dec⁻¹). This suggests that Ru‐RuO₂/C‐300A exhibited the most efficient electron‐transfer kinetics for OER among the samples, making it competitive with commercial RuO₂/C (79.8 mV dec⁻¹). Ru‐RuO₂/C‐300A also demonstrated excellent durability for OER, where after a 10 h chronoamperometric test at +1.5 V η_OER,10_ increased by only 30 mV to +270 mV after 10 h and to +310 mV after 24 h (Figure 4f and Figure S14, Supporting Information).

The exceptional durability of both HER and OER can be attributed to the strong electronic interactions at the Ru‐RuO₂ interface, which stabilize the active sites and mitigate degradation under harsh reaction conditions.^[^ ^16^ ^]^ These results underscore the robust nature of the catalyst, making it promising for long‐term applications in water electrolysis.

The Ru‐RuO₂/C‐300A sample even displayed an apparent OER performance in simulated seawater (1 m KOH + 0.5 m NaCl), alkaline seawater (seawater + 1 m KOH), and actual seawater (from the Natural Bridges State Beach in Santa Cruz, Figure S15, Supporting Information). Figure 4g shows the corresponding polarization curves, in comparison to that in 1 m KOH. The η_OER,10_ was estimated to be ≈+510 mV in actual seawater, but diminished markedly to only +270 mV in simulated seawater and +310 mV in alkaline seawater. This suggests a minimal activity of Ru‐RuO₂/C‐300A toward chlorine evolution reaction (CER) and the sample could be used as an effective OER catalyst even in high‐salinity electrolytes.^[^ ^46^, ^47^, ^48^ ^]^

From the above electrochemical measurements, Ru‐RuO₂/C‐300A can be seen to stand out as the best catalysts among the series toward both HER and OER in alkaline media. In fact, the performance is highly comparable to the leading results of relevant catalysts reported in the literature (Table S5, Supporting Information). Therefore, Ru‐RuO₂/C‐300A was used as the bifunctional catalyst for overall water splitting in 1 m KOH at a loading of 1 mg cm^−^^2^ on carbon paper. From the current‐voltage profiles in Figure 4h, the Ru‐RuO₂/C‐300A based cell required a voltage (E_10_) of only 1.43 V to achieve a current density of 10 mA cm^2^, which was 160 mV lower than that needed with a mixture of commercial 20% Pt/C and RuO₂/C (1.59 V). These results highlight the significant potential of Ru‐RuO₂/C‐300A as viable bifunctional catalysts for electrochemical water splitting.^[^ ^16^, ^17^, ^18^, ^19^, ^20^ ^]^

The remarkable bifunctional activities of Ru‐RuO₂/C‐300A can be attributed to the unique Ru‐RuO_2_ heterostructures produced by rapid heating of Ru(acac)_3_ with MIH. Prior research^[^ ^16^, ^17^, ^18^, ^19^, ^20^ ^]^ has shown that the formation of Ru‐RuO₂ M‐S heterojunctions significantly enhances the catalytic activity toward both HER and OER. This is because in HER, the Gibbs free energy for hydrogen adsorption is slightly reduced at the Ru‐RuO₂ interface, promoting optimal hydrogen adsorption and desorption; whereas in OER, the Ru‐RuO₂ interface optimizes the adsorption of oxygen intermediates and lowers the energy barrier for the formation of ^*^OOH intermediates. Such interactions can be facilitated by the optimal electron density of the Ru centers, leading to enhanced charge redistribution and electron transfer at the interface, as manifested in the above XPS measurements (Figure 2a, Table S2, Supporting Information).^[^ ^49^ ^]^

The fact that the metallic Ru and Ru^4+^ contents reached the maxima with Ru‐RuO₂/C‐300A is also in good agreement with the best HER and OER performances observed above (Table S3, Supporting Information). Such a unique structure was the result of ultrafast heating by MIH. For the sample prepared at a lower MIH current (e.g., Ru‐RuO_2_/C‐200A), the insufficient decomposition of Ru(acac)_3_ produced only amorphous Ru‐based clusters, whereas, at higher MIH currents (e.g., 400–600 A), evaporation of the precursor and samples diminished the Ru and RuO_2_ contents, leading to compromised electrocatalytic performance.

## 3 Conclusion

In summary, Ru‐RuO_2_/C heterostructure nanocomposites were prepared by rapid heating of Ru(acac)_3_ using MIH at controlled induction currents for 10 s. At low currents (e.g., 200 A), the insufficient thermal decomposition of Ru(acac)_3_ led to the production of largely amorphous ruthenium‐based clusters, whereas at higher MIH currents, the samples featured a hybrid structure where Ru and RuO₂ nanoparticles were in intimate contact. Among the series, Ru‐RuO₂/C‐300A possessed the highest contents of both metallic Ru and Ru^4+^ and hence exhibited the best HER and OER activity, requiring an η_HER,10_ of only −31 mV and η_OER,10_ of +240 mV in 1 m KOH. In addition, a comparable OER activity was observed in both simulated and alkaline seawater, suggesting minimal impacts of electrolyte salinity. Such a bifunctional activity is among the best of relevant catalysts reported in the literature and could be exploited for overall water splitting, where a low cell voltage of only 1.43 V was needed to achieve the current density of 10 mA cm^−2^, outperforming commercial 20% Pt/C and RuO₂/C mixtures by ≈160 mV. The catalysts also exhibited excellent durability, with minimal overpotential shifts after extended operation. These findings underscore the unique potential of Ru‐RuO₂ heterostructure composites as bifunctional electrocatalysts for efficient and stable electrochemical water splitting and offer a promising approach to sustainable hydrogen production from seawater.

## 4 Experimental Section

### Chemicals

Ruthenium(III) acetylacetonate (Ru(acac)_3_, 24.21%Ru, Engelhard), carbon paper (TGP‐H‐90, Toray), ruthenium(IV) oxide (RuO_2_, 99.5%, anhydrous, ACROS Organics), Pt/C (20 wt.%, Alfa Aesar), potassium hydroxide (KOH, 99%, Acros), and ethanol anhydrous (Fisher Chemicals) were used as received without any further treatment. Water was purified with a Barnstead Nanopure Waster System (resistivity 18.2 MΩ cm).

### Synthesis of Ru‐RuO_2_/C Nanocomposites

The Ru‐RuO_2_/C nanocomposites were prepared by using the MIH apparatus described previously.^[^ ^23^, ^24^, ^25^, ^26^, ^27^ ^]^ In brief, 40 mg of carbon black and 1 mL of ethanol were added into a 12 mL test tube and sonicated for 30 min. 4 mL of a 0.025 m Ru(acac)_3_ solution was added to the tube under sonication for another 30 min. After the solution was fully mixed with the carbon black, the tube was vacuum dried in an oven overnight at 60 °C. The obtained black powder was evenly loaded on a 2.5 cm × 2.5 cm × 0.2 mm iron plate covered with same‐size graphite paper (0.01 mm thick, to avoid contamination of the iron plate). The loaded plate was fixed on a fire brick with iron nails and sealed in a quartz tube, which was then purged with high‐purity argon gas for 15 min before being inserted into a four‐turn induction coil (5 cm in diameter). MIH synthesis was carried out at select induction currents (X = 200–600 A) for 10 s. After cooling down to room temperature, the obtained sample was washed with H_2_O and ethanol 5 times to remove excessive metal precursor until the supernatant was clear. The collected samples were denoted as Ru‐RuO_2_/C‐X.

### Characterization

TEM measurements were carried out with a FEI Tecni G2 scope operated at 200 kV. EDS‐based elemental mapping analyses were conducted with a JEM‐2100F instrument operated at 200 kV. XPS measurements were performed with a Thermo Scientific K‐alpha spectrometer. XRD patterns were obtained using a Bruker D8 Advance diffractometer with Cu K_α_ radiation (λ = 0.15 418 nm). Raman spectra were acquired with a Horiba Jobin Yvon LabRAM ARAMIS automated scanning confocal Raman microscope under 532 nm excitation. XAS measurements were conducted at 10 K using an Oxford liquid helium cryostat at beamline 4–1 of the Stanford Synchrotron Radiation Light source. The obtained XAS data were reduced, fitted, and analyzed with the RSXAP software.^[^ ^50^ ^]^ The Fourier Transform range was 3.5–12 for Ru K edges, while the fit range was 1.1–2.5 for Ru K edge. The theoretical functions for each pair (Ru‐C/O, Ru‐Ru) were calculated by WebAtoms.^[^ ^51^ ^]^

### Electrochemistry

Electrochemical measurements were conducted in 1 m KOH with a CHI 700E electrochemical workstation in a typical three‐electrode setup. The working electrode was a glassy carbon rotating disk electrode (RDE) with a surface area of 0.196 cm^2^, along with a graphite rod counter electrode and a Hg/HgO reference electrode. The reference electrode was calibrated against a reversible hydrogen electrode (RHE) and all potentials in the present study were referenced to this RHE. For the ink preparation, 5 mg of the catalysts obtained above were mixed with 200 µL of Nanopure H_2_O, 790 µL of ethanol, and 10 µL of Nafion under sonication for 30 min in an ice‐water bath. 10 µL of the ink and 5 µL of a 20% Nafion/IPA solution was drop cast onto the surface of the RDE evenly and dried in air (corresponding to a catalyst mass loading of 0.25 mg cm^−2^). Electrochemical impedance spectroscopy (EIS) tests were conducted with a Gamry Reference 600 instrument.

Full water splitting was performed in a two‐electrode configuration.^[^ ^52^ ^]^ Two pieces of graphite paper (1 cm × 2 cm) were cut for the anode and cathode. 100 µL of the catalyst ink (3 mg catalyst 60 µL H_2_O, 230 µL ethanol, and 10 µL Nafion) was loaded on a 1 cm × 1 cm area at a catalyst loading of 1 mg cm^−2^. All electrochemical measurements were repeated at least three times.

## Conflict of Interest

The authors declare no conflict of interest.

## Supporting information



Supporting Information

## Data Availability

The data that support the findings of this study are available from the corresponding author upon reasonable request.
