# Supplementary material for: Rapid Synthesis of Carbon‐Supported Ru‐RuO₂ Heterostructures for Efficient Electrochemical Water Splitting
Source: Adv Sci (Weinh). 2025 Jan 15;12(10):2414534. doi: 10.1002/advs.202414534 (PMC11904969; doi:10.1002/advs.202414534)
Supplement: Supplementary file 1 — Supporting Information [file ADVS-12-2414534-s001.pdf]

ADVANCED SCIENCE
Open Access

Supporting Information

for *Adv. Sci.*, DOI 10.1002/advs.202414534

Rapid Synthesis of Carbon-Supported Ru-RuO_2 Heterostructures for Efficient Electrochemical Water Splitting

*Dingjie Pan, Bingzhe Yu, John Tressel, Sarah Yu, Pranav Saravanan, Naya Sangoram, Andrea Ornelas-Perez, Frank Bridges and Shaowei Chen**

Supporting Information

Rapid Synthesis of Carbon Supported Ru-RuO_2 Heterostructures for Efficient Electrochemical Water Splitting

Dingjie Pan[a], Bingzhe Yu[a], John Tressel[a], Sarah Yu[a], Pranav Saravanan[a], Naya Sangoram[a], Andrea Ornelas-Perez[a], Frank Bridges[b], and Shaowei Chen[a,*]

[a] Department of Chemistry and Biochemistry, University of California, 1156 High Street, Santa Cruz, California 95064, United States

[b] Department of Physics, University of California, 1156 High Street, Santa Cruz, California 95064, United States

* E-mail: shaowei@ucsc.edu

List of Contents

- 15 Figures
- 5 Tables

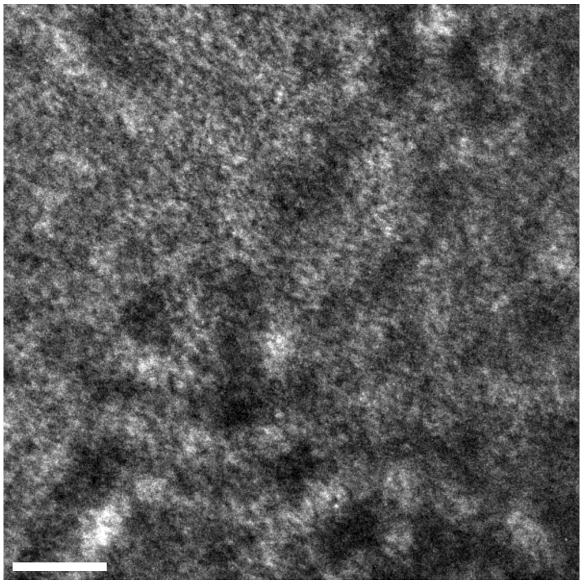

Figure S1. High-resolution TEM image of the Ru-RuO_2/C-200A sample.

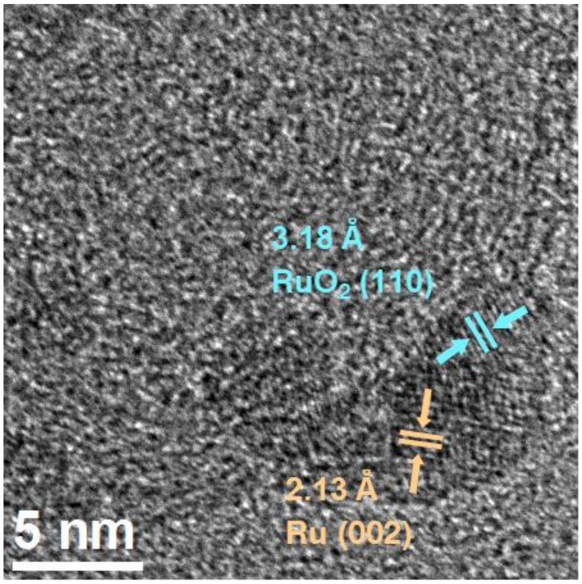

Figure S2. High-resolution TEM image of the Ru-RuO_2/C-400A sample.

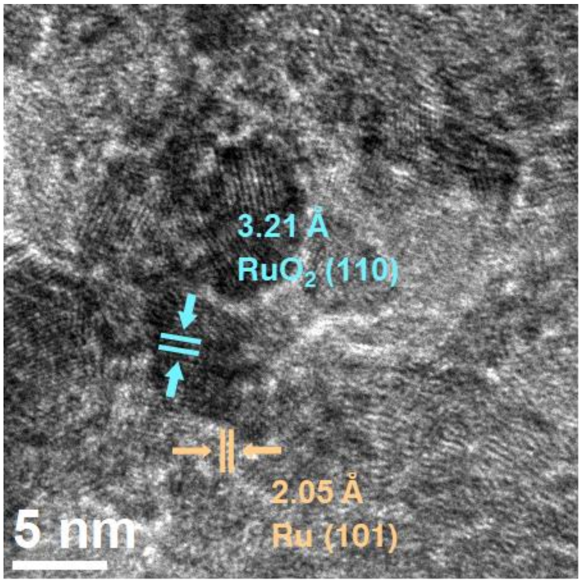

Figure S3. High-resolution TEM image of the Ru-RuO_2/C-500A sample.

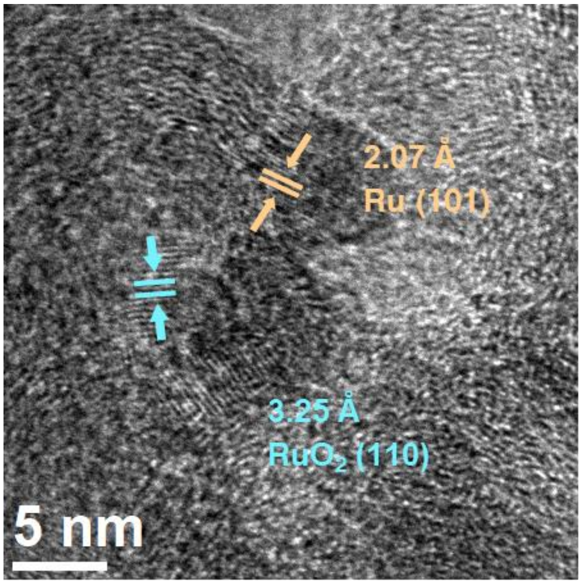

Figure S4. High-resolution TEM image of the Ru-RuO_2/C-600A sample.

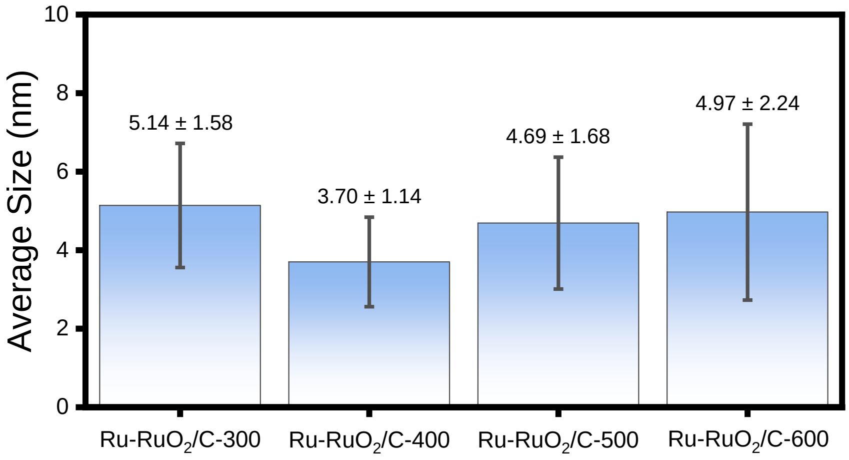

Figure S5. Average nanoparticle size of the Ru-RuO_2/C series.

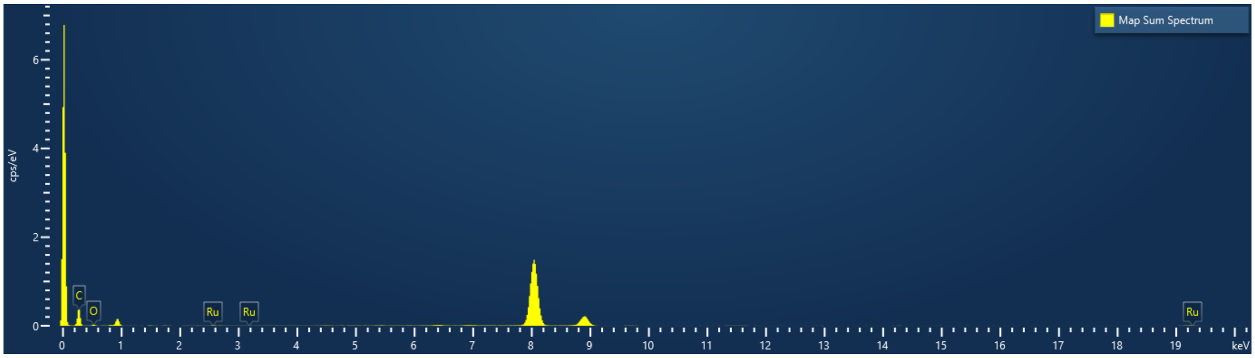

Figure S6. EDS spectrum of Ru-RuO_2/C-300A. The elemental compositions (C 89.16 wt%, O 2.59 wt%, and Ru 8.25 wt%) are in good agreement those obtained from XPS measurements (**Table S1**).

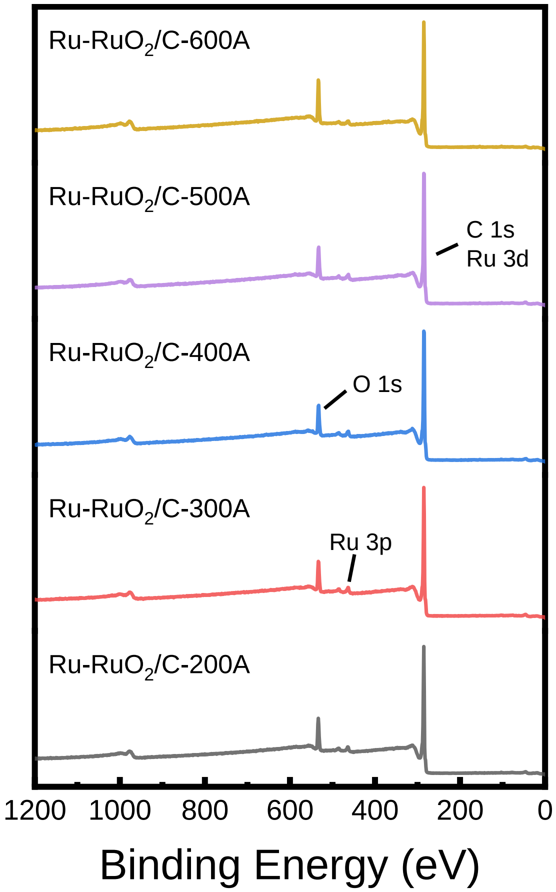

Figure S7. XPS survey spectra of the Ru-RuO_2/C series.

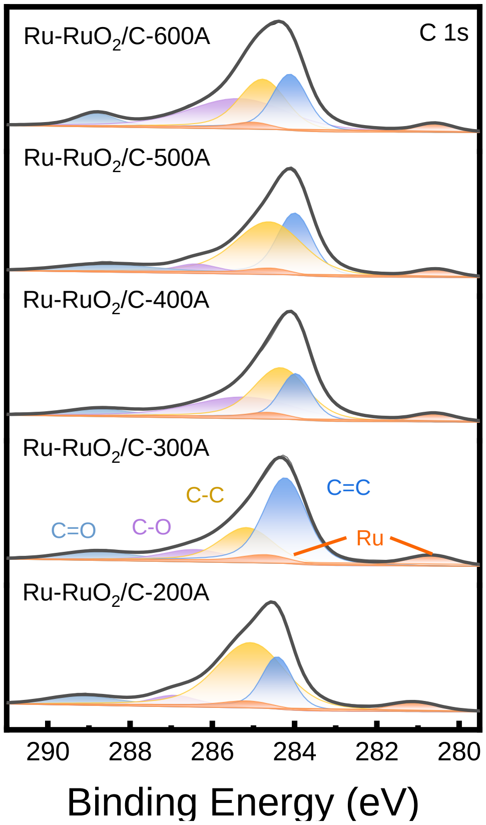

Figure S8. High-resolution XPS C 1s/Ru 3d spectra of Ru-RuO_2/C-X.

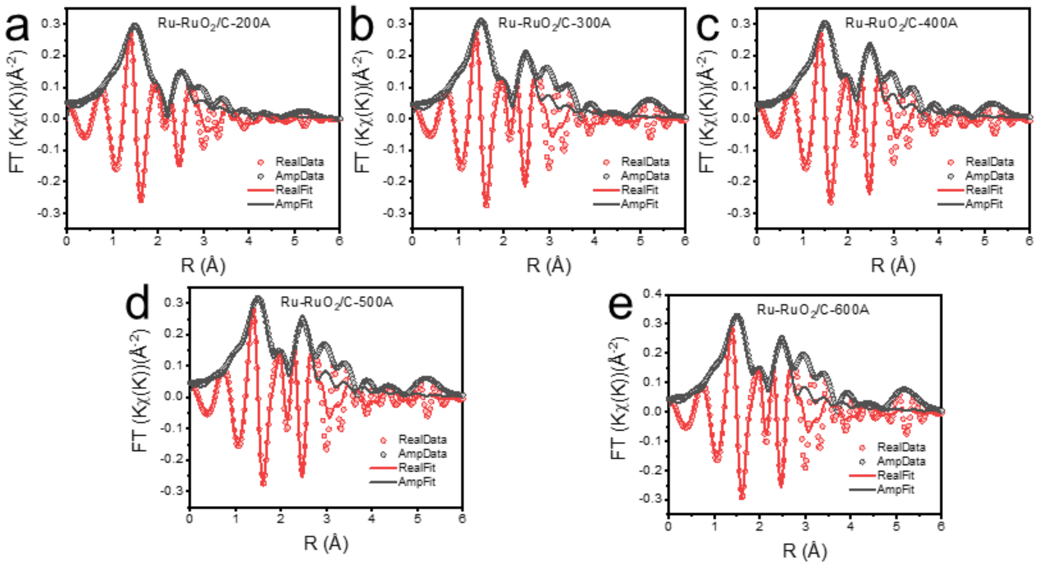

Figure S9. Fitting results of the Ru K edge EXAFS of the Ru-RuO_2/C samples.

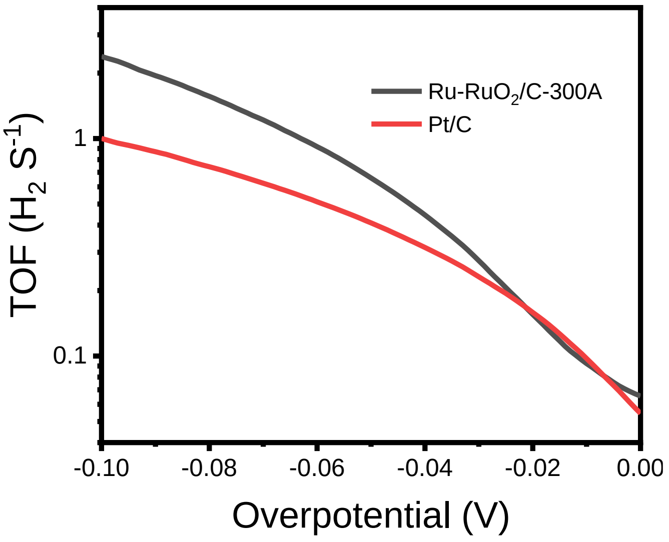

Figure S10. Comparison of the HER TOF of Ru-RuO_2/C-300A and commercial Pt/C.

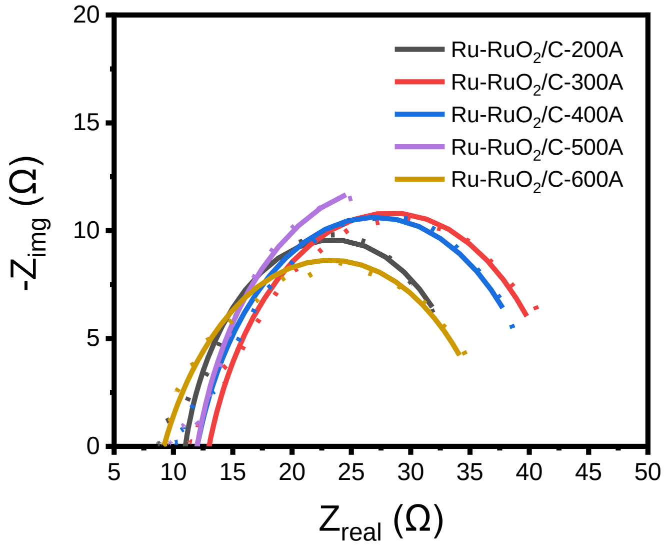

Figure S11. Nyquist plots at -50 mV for Ru-RuO_2/C- X samples, where the dashed lines represent the real data, and the solid lines indicate the fitting data with the equivalent circuit.

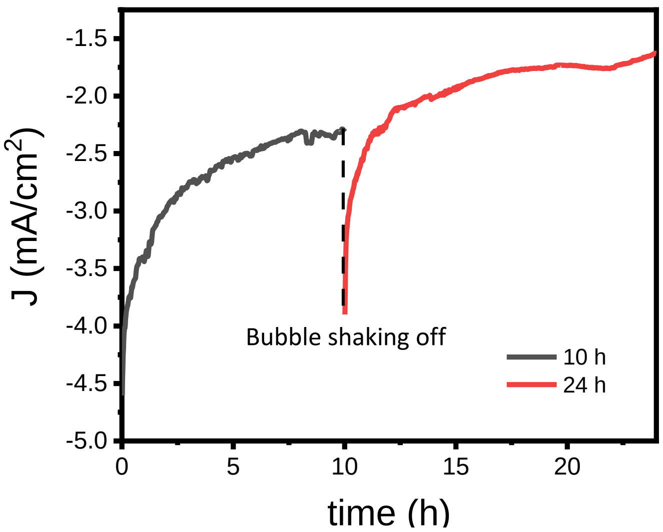

Figure S12. HER chronoamperometry curve of Ru-RuO_2/C-300A at -31 mV for 24 h. The diminishment of the current is mostly due to the large number of gas bubbles that block part of the electrode surface. Upon shaking off the gas bubbles, the current is recovered (red segment).

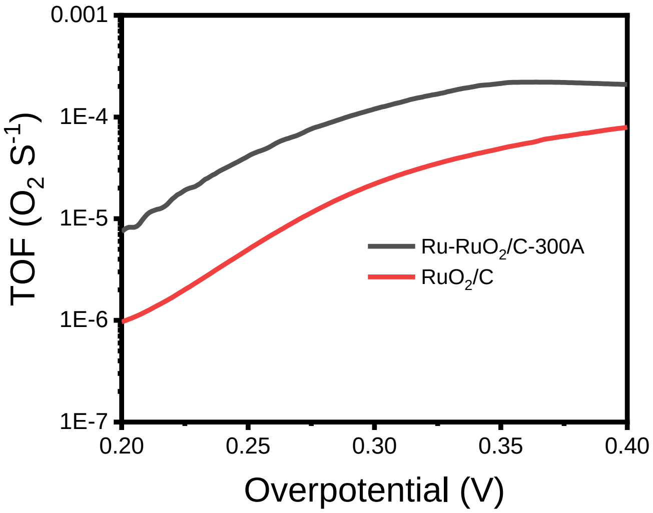

Figure S13. Comparison of the OER TOF of Ru-RuO_2/C-300A and commercial RuO_2/C.

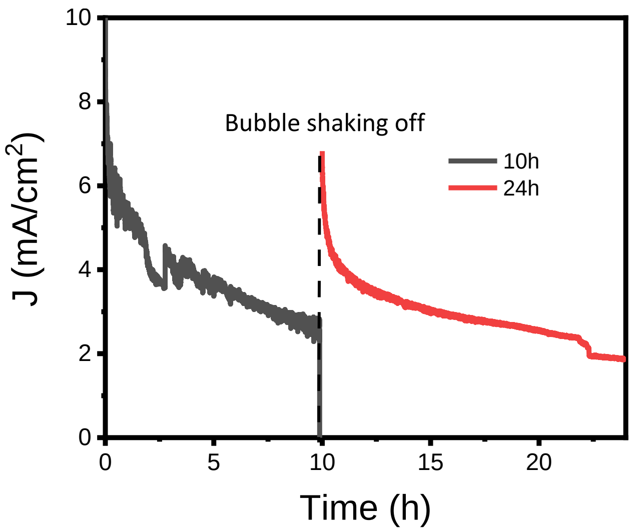

Figure S14. OER chronoamperometric curve of Ru-RuO_2/C-300A at 1.5 V for 24 h. The diminishment of the current is mostly due to the large number of gas bubbles produced that block part of the electrode surface. Upon shaking off the gas bubbles, the current is recovered (red segment).

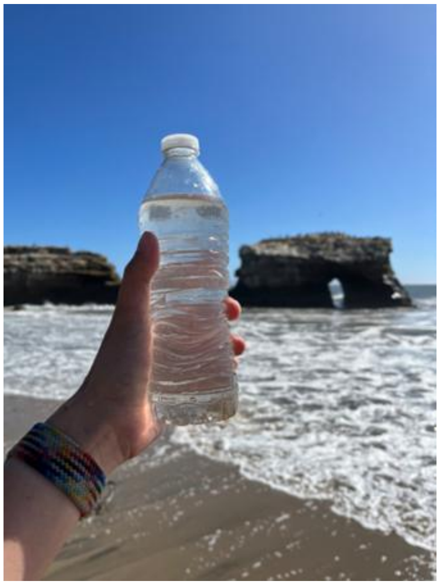

Figure S15. Seawater collected from Natural Bridges State Beach in Santa Cruz used as the saline water electrolyte for OER experiments.

Table S1. Elemental compositions estimated from XPS measurements.

	$Ru-RuO_2/C$-200A		$Ru-RuO_2/C$-300A		$Ru-RuO_2/C$-400A		$Ru-RuO_2/C$-500A		$Ru-RuO_2/C$-600A	
	at %	wt %	at %	wt %	at %	wt %	at %	wt %	at %	wt %
**C**	88.24	80.62	88.27	79.87	89.62	82.05	89.39	82.29	86.41	79.47
**Ru**	0.78	6.01	0.93	7.11	0.82	6.29	0.72	5.58	0.60	4.61
**O**	10.98	13.37	10.80	13.02	9.56	11.66	9.89	12.13	12.99	15.92

Table S2. Binding energies (eV) of the elemental components by deconvolution of the XPS data.

**Component**		$Ru-RuO_2/C$-200A	$Ru-RuO_2/C$-300A	$Ru-RuO_2/C$-400A	$Ru-RuO_2/C$-500A	$Ru-RuO_2/C$-600A
C 1s	Ru 5/2	281.09	280.67	280.59	280.57	280.59
	C=C	284.42	284.23	283.97	283.99	284.11
	C-C	285.06	284.67	284.33	284.57	284.77
	Ru 3/2	285.09	285.16	284.59	284.61	284.99
	C-O	286.93	286.44	285.18	286.4	285.32
	C=O	289.16	288.84	288.78	288.6	288.83
Ru 3p	Ru 3/2	462.31	461.77	461.84	461.78	461.94
	Ru^{4+} 3/2	464.56	463.94	464.26	464.17	464.54
	Ru^{4+} sat 3/2	467.54	466.75	467.25	466.99	467.75
	Ru 1/2	484.51	483.97	484.04	483.98	484.14
	Ru^{4+} 1/2	486.76	486.14	486.46	486.37	486.74
	Ru^{4+} sat 1/2	489.74	488.95	489.45	489.19	489.95
O 1s	Metal-O	530.07	529.88	529.67	529.61	529.53
	C=O	532.08	532.03	531.73	531.8	531.87
	C-O	533.54	533.51	533.21	533.28	533.3

Table S3. Elemental contents (at%) of metallic Ru and Ru^{4+} by XPS analysis.

**Species**	**Ru-RuO$_2$/C-200A**	**Ru-RuO$_2$/C-300A**	**Ru-RuO$_2$/C-400A**	**Ru-RuO$_2$/C-500A**	**Ru-RuO$_2$/C-600A**
Metallic Ru	0.44	0.52	0.47	0.41	0.36
Ru^{4+}	0.34	0.41	0.35	0.31	0.23

Table S4. Fitting results of the Ru K edge XAS spectra of Ru-RuO$_2$/C-X samples.

**Samples**	**Peak**	**σ^2 (Å^2)**	**Bond Distance (Å)**	**CN**
**Ru foil**	Ru-Ru	0.0711	2.680	12.00
**RuO$_2$**	Ru-O	0.0786	1.960	6.00
**Ru-RuO$_2$/C-200A**	Ru-C/O	0.0617	1.9695	4.182
	Ru-Ru (metal)	0.0580	2.6915	1.542
**Ru-RuO$_2$/C-300A**	Ru-C/O	0.0495	1.9644	3.747
	Ru-Ru (metal)	0.0562	2.6941	2.431
**Ru-RuO$_2$/C-400A**	Ru-C/O	0.0547	1.9604	3.816
	Ru-Ru (metal)	0.0519	2.6909	2.646
**Ru-RuO$_2$/C-500A**	Ru-C/O	0.0493	1.9604	3.744
	Ru-Ru (metal)	0.0448	2.6915	2.622
**Ru-RuO$_2$/C-600A**	Ru-C/O	0.0490	1.9582	3.867
	Ru-Ru (metal)	0.0456	2.6967	2.640

Table S5. Comparison of electrochemistry performance between Ru-RuO_2/C-300A and other Ru-based electrocatalysts in 1 M KOH

Electrocatalysts	$\eta_{HER,10}$ (mV)	$\eta_{OER,10}$ (mV)	Mass Loading (mg cm^{-2})	References
Ru-RuO_2/C-300A	-31	240	0.25	**This work**
Ru-RuO_2@NPC	-79	190	0.41	[1]
Ru-G/CC	-40	270	--	[2]
Co-SAC/RuO_2	-45	200	0.80	[3]
Ru-doped $Zn_3V_3O_8$	-70	260	2.00	[4]
RuO_2–Fe_2O_3	-239	399	0.19	[5]
Ru-TNTA	-41	349	--	[6]
Ru-RuO_2/Mn- MoO_2	-15	260	0.42	[7]
Ru@RuO_2-250	-32	182	0.26	[8]
$RuNi_7FeO_x(OH)_y$@NCA	-99	278	1.37	[9]

References

1. Wang, N.; Ning, S.; Yu, X.; Chen, D.; Li, Z.; Xu, J.; Meng, H.; Zhao, D.; Li, L.; Liu, Q.; Lu, B.; Chen, S., Graphene composites with Ru-RuO2 heterostructures: Highly efficient Mott–Schottky-type electrocatalysts for pH-universal water splitting and flexible zinc–air batteries. *Appl Catal B: Environ* **2022,** *302*, 120838.
2. You, M.; Du, X.; Hou, X.; Wang, Z.; Zhou, Y.; Ji, H.; Zhang, L.; Zhang, Z.; Yi, S.; Chen, D., In-situ growth of ruthenium-based nanostructure on carbon cloth for superior electrocatalytic activity towards HER and OER. *Appl Catal B: Environ* **2022,** *317*, 121729.
3. Shah, K.; Dai, R.; Mateen, M.; Hassan, Z.; Zhuang, Z.; Liu, C.; Israr, M.; Cheong, W. C.; Hu, B.; Tu, R.; Zhang, C.; Chen, X.; Peng, Q.; Chen, C.; Li, Y., Cobalt Single Atom Incorporated in Ruthenium Oxide Sphere: A Robust Bifunctional Electrocatalyst for HER and OER. *Angew Chem Int Ed* **2022,** *61* (4), e202114951.
4. Zhou, X.; Tang, X.; Xu, H.; Jiang, T.; Hu, K.; Qiu, H.-J.; Lin, X., Designing Ru-doped Zn3V3O8 bifunctional OER and HER catalysts through a unified computational and experimental approach. *Nanoscale* **2021,** *13* (41), 17457-17464.
5. Mosallaei, H.; Hadadzadeh, H.; Ensafi, A. A.; Mousaabadi, K. Z.; Weil, M.; Foelske, A.; Sauer, M., Evaluation of HER and OER electrocatalytic activity over RuO2–Fe2O3 nanocomposite deposited on HrGO nanosheets. *Int J Hydrogen Energy* **2023,** *48* (5), 1813-1830.
6. Liu, Y.; Wang, X.; Yang, M.; Li, Y.; Xiao, Y.; Zhao, J., Preparation of Ru-doped TiO2 nanotube arrays through anodizing TiRu alloys for bifunctional HER/OER electrocatalysts. *Nanoscale* **2023,** *15* (44), 17936-17945.
7. Xie, X.; Zhang, X.; Tian, W.; Zhang, X.; Ding, J.; Liu, Y.; Lu, S., Tri-functional Ru-RuO2/Mn-MoO2 composite: A high efficient electrocatalyst for overall water splitting and rechargeable Zn–air batteries. *Chem Eng J* **2023,** *468*, 143760.
8. Li, Z.; Zou, J.; Liang, T.; Song, X.; Li, Z.; Wen, J.; Peng, M.; Zeng, X.; Huang, H.; Wu, H., MOF-derived ultrasmall Ru@RuO2 heterostructures as bifunctional and pH-universal electrocatalysts for 0.79 V asymmetric amphoteric overall water splitting. *Chem Eng J* **2023,** *460*, 141672.
9. Huang, S.; Lu, J.; Wu, X.; Zhu, H.; Shen, X.; Cui, S.; Chen, X., Ru-promoted NiFe oxyhydroxide anchored on the hierarchical porous N-doped carbon aerogel: Electronic structures modulation for much enhanced OER/HER dual-functional characteristics. *Appl Catal A: Gen* **2023,** *664*, 119331.
